# Bilateral Congenital Retinal Macrovessel: A Case Report

**DOI:** 10.7759/cureus.33521

**Published:** 2023-01-08

**Authors:** Dalal Ismaeel, Soner Keskin, Foued Dallali

**Affiliations:** 1 Ophthalmology, King Hamad University Hospital, Muharraq, BHR

**Keywords:** foveal avascular zone, optical coherence tomography angiography, congenital anomaly, congenital retinal macrovessel, retina

## Abstract

Congenital retinal macrovessel (CRM) is a congenital anomaly where an aberrant vessel supplies both the macula on either side of the horizontal raphe. It is usually an incidental finding but may be associated with other retinal findings that may impact vision. We present an unusual case of bilateral congenital retinal macrovessel in a 40-year-old patient. The patient presented to the ophthalmology clinic for diabetic retinopathy screening and had no visual complaints. The patient underwent a complete ophthalmological examination including fundus imaging, fundus fluorescein angiography, and optical coherence tomography (OCT) imaging. Dilated fundus examination showed bilateral hypertensive retinopathy with optic disc collateral vessels and congenital retinal macrovessels. The multimodal imaging techniques that were used to confirm the finding indicated that the vessel is arterial.

## Introduction

Congenital retinal macrovessel (CRM), first described by Mauthner in 1869 [1], is a rare finding of an aberrant retinal vessel that crosses the macula and supplies or drains the retina inferior and superior to the horizontal raphe [2]. Later, it was classified as a form of arteriovenous malformation of the retina by Archer et al. [3]. This finding, while commonly incidental, has been seen in association with other pathologies such as branch retinal artery occlusion, macroaneurysm, telangiectasia, macular hemorrhage, and cavernous hemangioma [4-8]. More recently, an association with venous anomalies of the central nervous system has been proposed [9].

## Case presentation

**Figure 1:**
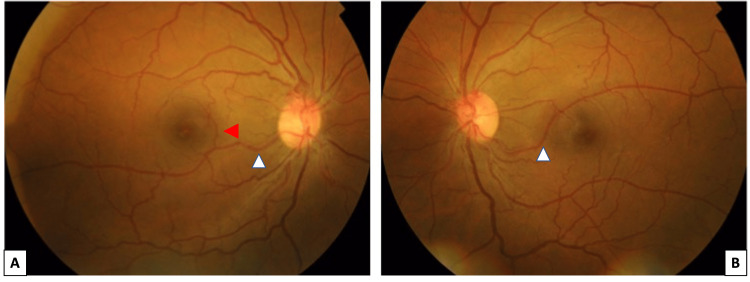
Color fundus imaging of the right (A) and left (B) eyes. White arrowheads pointing to the congenital retinal macrovessels and the red arrowhead pointing to the branch crossing the horizontal raphe in the right eye.

A 40-year-old male who is known to have diabetes mellitus, dyslipidemia, and hypertension for the last eight years presented to the ophthalmology clinic for diabetic retinopathy screening. He had no previous ocular history. On examination, his best corrected visual acuity was 6/6 in both eyes. Slit lamp examination of the anterior segment was normal in both eyes. Dilated fundus examination revealed bilateral grade 2 hypertensive retinopathy with optic disc collateral vessels. In both eyes, an aberrant vessel was found originating from the optic nerve head and traversing the papillomacular bundle inferotemporally. In the right eye, the vessel gives off a branch that crosses superiorly to supply the fovea, whereas in the left eye, the entire vessel crosses the horizontal raphe as shown in the fundus image below (Figure 1). The caliber of the vessel was similar to that of a retinal artery in both eyes.

**Figure 2:**
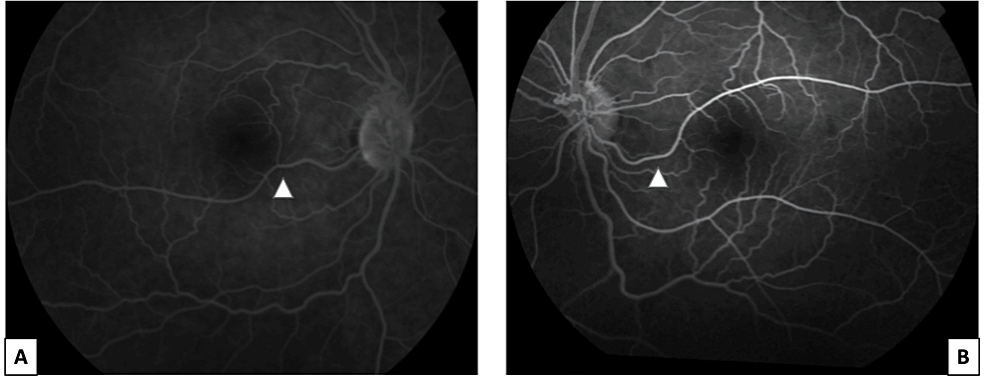
Fundus fluorescein angiography of the right (A) and left (B) eyes. White arrowheads pointing toward the congenital retinal macrovessels.

**Figure 3:**
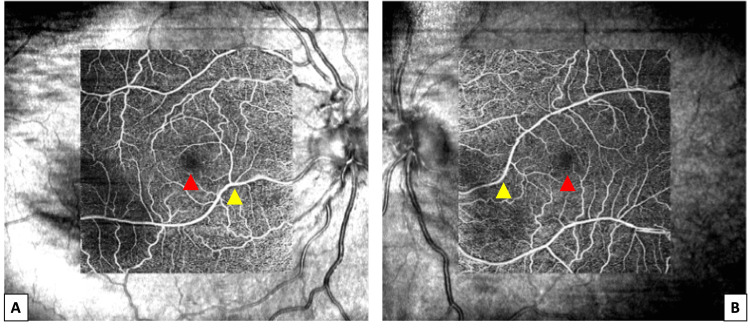
OCT-A images of the right (A) and left (B) eyes. Red arrowheads pointing toward FAZ and yellow arrowheads pointing toward the congenital retinal macrovessels. FAZ, foveal avascular zone; OCT-A, optical coherence tomography angiography.

Fundus fluorescein angiography showed the filling of the vessel early in the arterial phase, and no leakage was noted (Figure 2). Optical coherence tomography (OCT) and optical coherence tomography angiography (OCT-A) scans were obtained using Zeiss Cirrus HD-OCT (Zeiss, Oberkochen, Germany). In both eyes, no enlargement of the foveal avascular zone (FAZ) was observed (Figure 3).

## Discussion

Congenital retinal macrovessel is an abnormal macular vessel with distribution above and below the horizontal raphe [1,2]. The term retinal venous malformation by Pichi et al. [9] has been suggested in order to highlight the potential presence of cerebral venous malformations found in association with this condition. In their cross-sectional multicenter study, all cases were unilateral, and the presence of cerebral venous malformation was established in 24% of the patients, with 83% being ipsilateral to the CRM and 75% involving the frontal lobe [9]. In our case, the patient did not consent to have a brain magnetic resonance imaging (MRI) done; therefore, the presence of a cerebral venous malformation could not be determined. Most cases of congenital retinal macrovessel are found incidentally with no impact on visual acuity suggesting a possible underestimation of its prevalence. Rarely, it may be found in conjunction with vitreous hemorrhage, foveal cyst, serous retinal detachment, branch retinal artery occlusion, macroaneurysm, telangiectasia, macular hemorrhage, or cavernous hemangioma [4-12]. The occurrence of the aforementioned associations can result in the deterioration of visual function. Fundus fluorescein angiography findings include early filling with delayed emptying of the vessel and the enlargement of FAZ occasionally. Other findings seen on angiography include dilated capillaries, areas of capillary non-perfusion, and dye leakage [9,13,14]. To our knowledge, no case of bilateral involvement has been reported.

## Conclusions

In conclusion, we report the case of bilateral congenital retinal macrovessel, which we believe is arterial in origin. This finding was observed incidentally as the patient was asymptomatic. Further investigation with imaging modalities highlighted the presence of this retinal vascular anomaly bilaterally. No macular thickening or FAZ enlargement was observed on OCT and OCT-A imaging, respectively.
